# Comparing Simultaneous Scalp EEG Recordings from the OpenBCI Cyton and Brain Products BrainAmp

**DOI:** 10.3390/s26041153

**Published:** 2026-02-11

**Authors:** Alessandro D’Amico, Virginia R. de Sa

**Affiliations:** 1 Department of Cognitive Science, University of California, San Diego, CA 92093, USA; adamico@ucsd.edu; 2 Halıcıoğlu Data Science Institute, University of California, San Diego, CA 92093, USA; 3 Institute for Neural Computation, University of California, San Diego, CA 92093, USA

**Keywords:** EEG, ERP, P300, P3b, N400, ERN, low-cost neuroimaging, open-source, OpenBCI, cyton, brain products, BrainAmp

## Abstract

Portable, affordable electroencephalography (EEG) amplifiers could enable neuroscience-scale data collection. The open-source OpenBCI Cyton shows promise in this regard, but remains undervalidated for cognitive neuroscience ERP experiments. We simultaneously recorded eight scalp electrodes with both Cyton and gold-standard Brain Products BrainAmp amplifiers across P3b-, ERN-, and N400-eliciting tasks. Five healthy volunteers completed visual oddball (P3b), flankers (ERN), and word association (N400) tasks. We quantified within-subject signal similarity using Pearson $r^{2}$, mean absolute error (MAE), mean arctangent absolute percentage error (MAAPE), and within-component window mean and standard deviation. Cyton signals showed $r^{2}$ = 97–100%, MAE ≈ 1 µV, and MAAPE ≈ 20% with BrainAmp signals at ERP sites of interest. No significant differences emerged in mean amplitudes within ERP component windows across amplifiers, though standard deviations differed significantly. These results demonstrate that the Cyton records highly similar but not identical scalp EEG as research-grade equipment. This first multi-subject, concurrent scalp EEG validation across multiple ERP components validates the Cyton for cognitive neuroscience and supports broader adoption of affordable open-source tools.

## 1. Introduction

Neuroimaging of humans has provided key insights into the role of the nervous system in cognition. For most of this field’s history, imaging has been confined to specialized university laboratories that rely on prohibitively expensive recording equipment. In the last decade, technologies such as functional near-infrared spectroscopy (fNIRS) and scalp electroencephalography (EEG) have begun to move beyond the laboratory setting. Consequently, researchers have become interested in “naturalistic neuroimaging,” that is, recording neural data in ecologically valid environments. Low-cost EEG systems have become increasingly available and popular, bringing with them the potential for naturalistic neuroimaging. Furthermore, low-cost systems may enable the collection of significantly larger volumes of neural data from more diverse populations. Before such endeavors can be rigorously pursued, however, it is necessary to validate these low-cost systems against well-established, “gold-standard” equipment. This paper validates the OpenBCI Cyton, a popular, open-source, low-cost EEG system [1]. The Brain Products BrainAmp served as the “gold-standard” amplifier for comparison with the Cyton. The Cyton and Brain Products BrainAmp [2] simultaneously recorded signals from eight EEG electrodes. Data from both amplifiers were subsequently compared to assess the Cyton’s ability to record high-quality data.

According to a systematic review of low-cost EEG systems by Sabio et al. [3], OpenBCI hardware appears in 67 peer-reviewed articles, making them the third most prominent low-cost EEG manufacturer behind NeuroSky (225 publications) and Emotiv (595 publications). However, OpenBCI amplifiers attract particular researcher interest due to their open design. In addition to open-source hardware, firmware, and software, OpenBCI amplifiers accept various electrode types. Researchers can place these electrodes arbitrarily, providing customizability for recording montages. In contrast, NeuroSky and Emotiv consumer-grade products do not allow montage modification. For many studies, especially event-related potential (ERP) studies, researchers need to place electrodes at specific sites. Given OpenBCI’s prominence and customizability, we selected it as an ideal validation candidate. Despite its popularity, few studies have systematically validated the Cyton. Rashid et al. [4] compared the Cyton to a NuAmps amplifier, but collected participant data from each amplifier during separate recording sessions (a sequential rather than simultaneous method). Frey [5] performed simultaneous multi-electrode recordings from the Cyton and g.tec’s g.USBamp, but only on a single subject. Similarly, Knierim et al. [6] simultaneously recorded from the Cyton and MBrainTrain’s Smarting Mobi 24, but placed electrodes around the ear rather than on the scalp. Thus, this study provides the first multi-subject, simultaneous comparison of the OpenBCI Cyton’s scalp EEG recording ability.

This study compares EEG data from tasks designed to elicit three commonly studied ERP components: the P3b (for an overview, see [7]), elicited by an active visual oddball task Kutas et al. [8]; the N400 (for an overview, see [9]), elicited by an active word-pair association task Holcomb and Neville [10]; and the error-related negativity (ERN; for an overview, see [11]), elicited by a flankers task [12,13]. A meta-analysis of 98 ERP components [14] ranks the P300 as the most published ERP component, the N400 third, and the ERN eighth. We selected these tasks and components to appeal to a broad range of ERP researchers. Kappenman et al.’s ERP CORE [15] has extensively validated these paradigms. This study extends ERP CORE by using identical task parameters, ERP windowing, and similar analyses. These similarities enable comparisons between our novel data and ERP CORE’s public dataset to assess general recording quality. A secondary goal of this study is to advance ERP CORE’s mission of open, reproducible ERP research. To achieve this, we are publishing the experiment software, analysis code, and data as open source.

## 2. Materials and Methods

This study was approved by the University of California, San Diego Institutional Review Board (UCSD IRB). All participants provided written informed consent.

### 2.1. Participants

We collected data from 5 participants (4 female and 1 male), all graduate or undergraduate students at UCSD. Participants volunteered their time without pay, course credit, or other compensation. We recorded no demographics, medication, sleep, or caffeine history. All participants verbally confirmed normal or corrected vision, no color blindness, and no personal or family history of epilepsy. Participants sat in a sturdy, cushioned wooden chair and were instructed to remain as still as possible during recordings while fixating on the screen center. Written per-task instructions appeared on the presentation monitor. After reading instructions, participants completed 10 monitored practice trials before each task. Each task included break periods between blocks for blinking, stretching, and adjusting position. We excluded participant “BC002” from P300 analyses because 50% of their trials were rejected (see Table 1). Their data were included in other ERP analyses. Due to the small sample size, this study focuses primarily on fully within-subject analyses. The only across-subject analyses examine noise histograms of the recordings (see Section 4.1 for details).

### 2.2. Stimuli and Tasks

All tasks were programmed using the Godot game engine (version .NET 4.1.3) [16] and followed the parameters specified by ERP CORE [15]. Godot .NET enabled the C# version of the Lab Streaming Layer protocol (LSL; [17]), which sent event markers to synchronize behavioral and neural data. Participants completed tasks on a Windows 10 machine with stimuli presented on a Dell P992 cathode-ray tube monitor (75 Hz refresh rate). Two photodiodes on the monitor sent signals to both amplifiers to synchronize stimulus events.

We randomized the task order within each participant using a random number generator. Participants completed the five-arrow variation in the Eriksen flanker task [12,13], an active visual oddball task using the first five letters of the alphabet adapted from Kutas et al. [8], and an active word-pair association task adapted from Holcomb and Neville [10] that uses only “legal” English words for both primes and targets. For the oddball and word association tasks, we counterbalanced response keys between subjects (up or down keys on a keyboard). Each task used a gray background with a constant white fixation circle at the screen center. Participants responded with their dominant hand (right *n* = 4; left *n* = 1). See Supplemental Methods for task-specific parameters. Since we analyzed response-locked ERPs from the flanker task, we removed trials with multiple responses. We retained double-response trials for the stimulus-locked visual oddball and word association tasks. See Supplemental Methods for task parameter details.

### 2.3. EEG Recording

**Figure 1:**
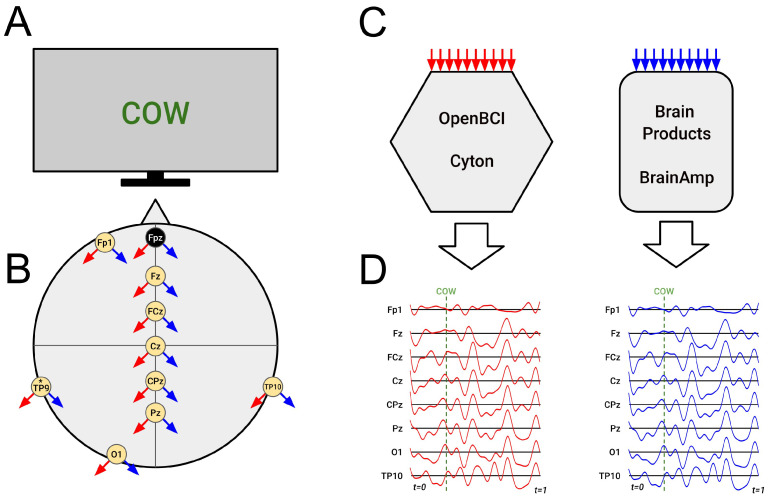
Simplified diagram of simultaneous electroencephalography (EEG) recording to two amplifiers. (**A**) The subject’s monitor displaying a stimulus, in this case the word “COW” from the word-pair association task.(**B**) Electrodes on the modified 10-10 system showing signals split in two directions. Each electrode was split to the OpenBCI Cyton (red arrows) and Brain Products BrainAmp (blue arrows) including the online reference electrode (TP9) as well as the ground/driven right leg electrode (Fpz). (**C**) Each electrode terminated onto both amplifiers. (**D**) EEG data was then amplified and digitized by both amplifiers independently. LSL streams were created from the digitized EEG data of both amplifiers and were synchronized alongside LSL marker streams from the task using LabRecorder. Data were subsequently digitally resampled then aligned via cross-correlation in order to make direct comparisons across amplifiers.

We simultaneously recorded continuous EEG data using the Cyton produced by OpenBCI Inc. (Brooklyn, NY, USA) and BrainAmp produced by Brain Products GmbH (Gilching, Germany). We placed passive (non-preamplified) Ag/AgCl electrodes on a custom mesh cap manufactured by Wuhan Greentek Pty. Ltd. (Wuhan, China) We used ten channels from the International 10/20 System (Fpz, Fp1, Fz, FCz, Cz, CPz, Pz, O1, TP9, TP10), with Fpz as the ground and TP9 as the online reference. We gelled electrodes using SuperVisc (Brain Products) and connected each via female touchproof (DIN 42802) connectors through custom Y-splitter cables (see Figure 1). We attached touchproof connectors to the Cyton via touchproof male to DuPont female adapters and to the BrainAmp via Brain Products’ Electrode Input Box. Both amplifiers applied unmodified hardware anti-aliasing filters during digitization (Cyton: 250 Hz; BrainAmp: 1000 Hz). We acquired Cyton data using OpenBCI GUI (version Beta 6.0.0) and BrainAmp data using Brain Vision Recorder (version 1.25.024), creating synchronized LSL streams via LabRecorder (version 1.16.4). All impedances were below 80 kΩ per OpenBCI GUI. We configured the Cyton with 1 ms FTDI buffers to increase temporal consistency [6].

### 2.4. Signal Processing

Signal processing was conducted in Python 3.12 using a custom toolbox utilizing NumPy 2.0.2 [18] and SciPy 1.14.1 [19]. As an initial preprocessing step, we linearly interpolated Cyton and BrainAmp data to their respective sampling rates using the Python library traces (version 0.6.1). This ensured evenly sampled data streams for accurate filtering and subsequent signal alignment. BrainAmp signals were then downsampled to 250 Hz to match the Cyton.

#### 2.4.1. Filtering

After interpolation and BrainAmp downsampling, we filtered both continuous EEG streams using noncausal finite impulse response (FIR) Hamming window filters. We created and applied separate high- and low-pass filter coefficients following recent systematic comparisons of preprocessing effects on EEG data [20,21], as well as the parameters determined by Kappenman et al. [15]. We used a 2751-tap high-pass filter with a cutoff of 0.3 Hz and a −6 dB cutoff frequency of 0.15 Hz alongside a 67-tap, 50 Hz cutoff (−6 dB at 56.25) low-pass filter. We selected a 50 Hz low-pass cutoff to minimize temporal smoothing of EEG data while attenuating 60 Hz line noise.

#### 2.4.2. Signal Alignment

We aligned continuous BrainAmp and Cyton streams by maximizing the cross-correlation of the average of all eight amplifier-specific channels. We computed optimal cross-correlation lags using a sliding window approach with 20 s discrete windows, storing the cross-correlation between amplifiers. After processing the entire continuous stream, we calculated the mode lag and used it to align the two recordings. During epoching, we further aligned BrainAmp and Cyton signals trial-by-trial using cross-correlation.

The experiment was designed to use photosensors simultaneously recorded by both amplifiers for synchronization. Unfortunately, the Cyton failed to accurately record photosensor signals via “Analog Read” functionality in the OpenBCI GUI (see Section 4.1), requiring the use of the cross-correlation approach. We conducted alignment on each recording independently. The average lag between the BrainAmp and Cyton streams was 31 ms (SD = 21 ms). These lags showed no systematic relation to recordings and likely resulted from LabRecorder. Cross-correlation provides good but imperfect synchronization, as discussed in Section 4.1.

#### 2.4.3. Re-Referencing and Artifact Rejection

EEG data were then re-referenced to the average of TP9 and TP10 within each amplifier. Data were epoched (binned) and baseline-corrected using the windows recommended in ERP CORE. ERPs in the oddball and word association task were stimulus-locked, and they were response-locked for the flankers task. Note that the flankers ERPs were baseline-corrected using the response onset and not the stimulus onset in order to match ERP CORE’s procedures. Stimulus-locked events were epoched relative to LSL events and further locked to the photosensor recorded by the BrainAmp, while response-locked events were epoched relative to LSL marker onsets alone. Simple-voltage thresholding was then performed on all epochs using all channels, with a threshold of 100 µV. Only points spanning the start of the baseline to the end of the component window of interest were examined for artifacts. Any epoch failing this procedure was rejected from analysis. Finally, each individual epoch went through a cross-correlation alignment process to further synchronize Cyton and BrainAmp streams.

### 2.5. Analysis

All primary analyses were conducted on data without epoch-level detrending. We also performed all analyses with linear detrending using the SciPy function signal.detrend(). Linear detrending systematically improved condition classification in a study using ERP CORE data [21]. We added detrending after observing substantial voltage drift over time in continuous Cyton (but not BrainAmp) data. Although the high-pass filter adequately removes this trend, we compare detrended and non-detrended results in Supplemental Methods.

#### 2.5.1. ERP Component Quantification

ERP components were quantified as the baseline-corrected mean of a window of interest. Epoch, baseline, and component windows were identical to those recommended in ERP CORE [15]. Measurements were taken at a single channel, also defined in ERP CORE’s methodology. We compared the fidelity to which the Cyton records these ERP components relative to the BrainAmp using both the means and standard deviations of these windows. Dependent sample *t*-tests were conducted within individuals to determine if there were statistically significant differences between BrainAmp and Cyton recordings. Effect sizes (Cohen’s d) were also calculated and reported.

#### 2.5.2. Signal Similarity Analysis

One-sided Pearson coefficients of determination ($r^{2}$) were computed as scale-invariant measurements of similarity across BrainAmp and Cyton data. Values were computed within each subject, channel, and trial. Each individual timepoint of the trial was used as a sample, with the Cyton data serving as the dependent variable, and BrainAmp data serving as the independent variable. This procedure thus measures the within-trial temporal correlation of both amplifiers. Correlation values, including coefficient *r* and significance *p*, were computed with the SciPy function stats.pearsonr(), with the alternative hypothesis set to “greater”, as high correlation between the signals was hypothesized. Correlation was computed on a window spanning the entire epoch. These values were averaged across trials within participants and reported in Table 2.

Additionally, one-dimensional canonical correlation analysis (CCA) loadings were learned using sci-kit learn’s cross_decomposition.CCA() to project data from all channels to a single dimension that could be correlated. For each trial, CCA loadings were trained using an average of three random trials excluding the one being analyzed. BrainAmp and Cyton data from the trial were then projected onto the trained loadings, and these projected signals were correlated using the one-channel method described previously. This procedure was repeated for 5 folds, and the average $r^{2}$ of these folds was stored per trial. Finally, values across all trials were averaged within participants and are reported in Supplementary Materials.

Correlation fails to capture scale-dependent similarity between signals. We computed mean absolute error (MAE) and mean arctangent absolute percentage error (MAAPE; [22]) between BrainAmp and Cyton signals. Like the correlation analysis, we computed these metrics within trials between amplifiers and averaged them for reporting. MAE equals the mean absolute difference between signals. We computed MAE on within-channel, within-condition BrainAmp−Cyton difference waves, matching the correlation analysis. MAAPE, on the other hand, provides relative error robust to near-zero values common in EEG data:

(1) $$\mathrm{MAAPE}=\frac{1}{N}\sum_{t=1}^{N}\arctan\left(\left|\frac{A_{t}-F_{t}}{A_{t}}\right|\right),$$

where $A_{t}$ = BrainAmp (actual) and $F_{t}$ = Cyton (forecast) signals [22].

#### 2.5.3. ERP Noise Quantification

**Figure 2:**
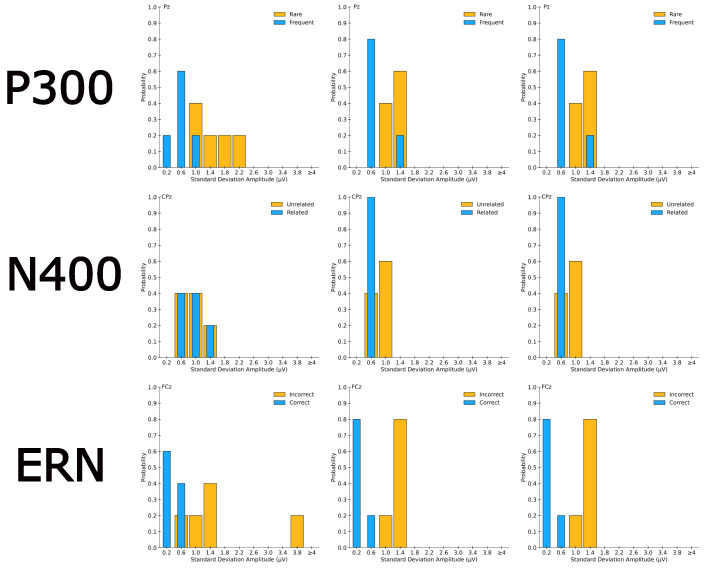
Quantification of ERP noise. Each histogram shows noise within the ERP measurement window (see Section 2; Figure 3 from Kappenman et al. [15]). Rows represent ERP components (top to bottom: P300, N400, ERN). (**Left**) ERP CORE noise from mode of 5000 *n* = 5 bootstraps. (**Middle**) BrainAmp noise from all five subjects. (**Right**) Cyton noise from all five subjects.

Correlational analyses comparing BrainAmp and Cyton data are susceptible to noise. Kappenman et al. provide a method for quantifying noise within each ERP window using “plus-minus averaging” [15]. We computed standard deviations from the plus–minus average within each ERP window for every component and subject. We visualized results using histograms (see Figure 2) and compared them to re-analyzed ERP CORE data (leftmost column of Figure 2). We used Mann–Whitney U tests to compare distributions (BrainAmp vs. ERP CORE and Cyton vs. ERP CORE) with one-tailed tests examining whether Cyton or BrainAmp noise exceeded ERP CORE noise. Unlike prior within-subject analyses, this analysis was across subjects. In order to compare ERP CORE’s data to our *n* = 5 sample, ERP CORE data from all 40 subjects were bootstrapped with an *n* = 5. Histograms were created for 5000 unique iterations of these *n* = 5 selections, and the mode distribution was compared to distributions from the Cyton and BrainAmp.

## 3. Results

### 3.1. ERP Components

Dependent-sample *t*-tests were conducted within subjects to assess whether ERP amplitudes differed between BrainAmp and Cyton signals in each condition. Bonferroni correction was applied across the full family of tests by dividing the nominal alpha of 0.05 by 20, reflecting five subjects, two conditions, and two dependent variables (mean and standard deviation of each component).

#### 3.1.1. ERP Window Means

**Figure 3:**
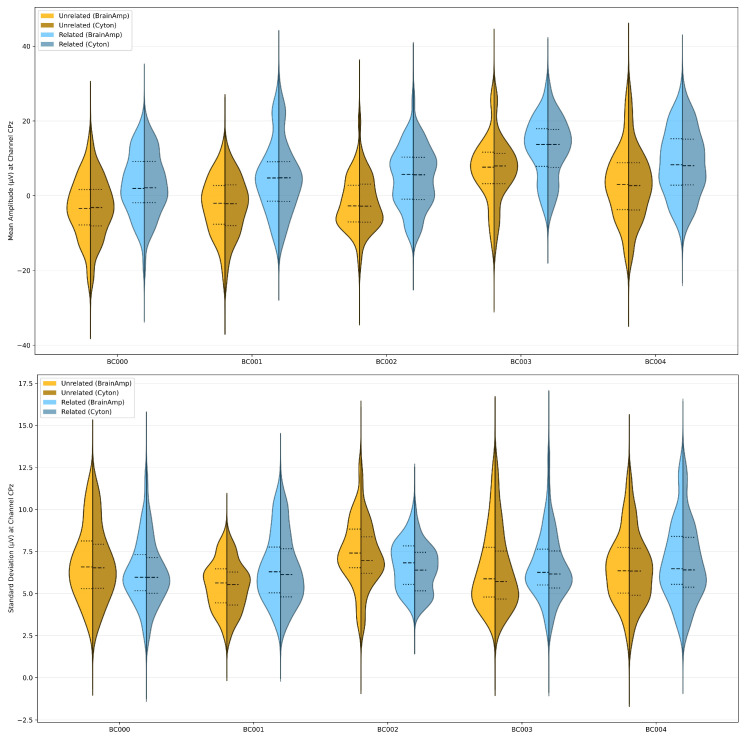
Violin plots of mean (**top**) and standard deviation (**bottom**) of ERP window data from the word association task. Both metrics were computed within each ERP window of interest and distributed across trials per subject. Gold plots represent the unrelated condition; blue plots represent the related condition. Light colors show BrainAmp recordings; dark colors show Cyton recordings. First and third quartiles (dotted lines) appear within each half of the asymmetrical violin plots alongside the mean (dashed lines). Note that tails may not accurately reflect variance due to rendering artifacts. See Supplemental Materials for visual oddball and flanker task plots.

For the visual oddball task, no subjects showed significant differences between amplifiers. For the word association task, subject “BC003” had significant differences in both conditions (unrelated: p<0.001, d=−0.49; frequent: p<0.0001, d=−0.51), while subject “BC004” showed differences only in the unrelated condition (p<0.002, d=−0.41). For the flanker task, significant differences appeared only in the correct-response condition for subjects “BC000” (p<0.0005, d=−0.19) and “BC001” (p<0.00001, d=0.25). See Figure 3 for a visual representation of these differences in the word association task.

#### 3.1.2. ERP Window Standard Deviations

We examined distributions of within-window standard deviations similarly to the component means. Unlike the means, significant differences appeared across all subjects and tasks. Results appear in Table 3 and can be visualized for the word association task in Figure 3.

### 3.2. Signal Similarity

**Figure 4:**
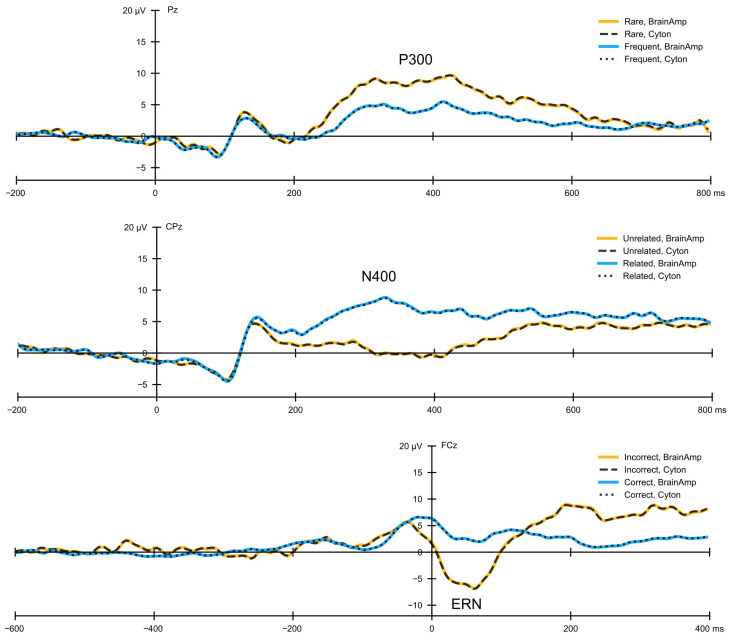
Grand-average ERPs for each task. Data were simultaneously recorded from two amplifiers (Cyton and BrainAmp) at the same electrode sites. Each row represents data from a different task; row 1 is from the visual oddball task, row 2 is from the word association task, and row 3 is from the flankers task. In each plot, the dashed and dotted lines represent data recorded from the low-cost Cyton, while the colored solid lines represent data recorded from the research-grade BrainAmp. Note: Positive is plotted up to match the style of plots presented in the ERP CORE publication [15].

Cyton signals explained 97–100% of BrainAmp signal variance ($r^{2}$) at all sites of interest across subjects. Mean absolute error averaged 0.79 µV across subjects and tasks. MAAPE averaged 0.2 across all subjects and tasks (see Table 2). This trend held true across all electrodes as well as the 1D CCA-projected data (see Supplementary Materials). All correlation analyses were strongly statistically significant even after correcting for the 60,804 comparisons (trials × channels × 2; Bonferroni-corrected $p<8.22\times 10^{-7}$) except for a single comparison. Signal similarity is visualized with grand average ERPs in Figure 4.

### 3.3. ERP Noise Quantification

The noise histograms were identical between Cyton and BrainAmp recordings. One-tailed Mann–Whitney U tests show that neither the BrainAmp nor the Cyton noise histograms are greater (i.e., noisier) than the ERP CORE noise histograms. When comparing BrainAmp and Cyton histograms to the mean ERP CORE distribution, incorrect ($U_{Cyton}=U_{BrainAmp}=26.0$) and correct ($U_{Cyton}=U_{BrainAmp}=60.5$) trials of the flankers task, related ($U_{Cyton}=U_{BrainAmp}=36$) and unrelated ($U_{Cyton}=U_{BrainAmp}=44.0$) trials of the word association task, and rare ($U_{Cyton}=U_{BrainAmp}=52.0$) and frequent ($U_{Cyton}=U_{BrainAmp}=52.5$) trials of the oddball task show no significant differences (p>0.59 in all tests). These findings replicate when looking at distributions from the 5000 permutations of 5 subjects or the averages from all 40 ERP CORE subjects (see Supplementary Materials).

### 3.4. Artifact Rejection, Linear Trend, and Signal Synchronization

Trial rejection rates varied across tasks and conditions. The visual oddball task showed the highest drop rate (20.71%), followed by word association (6.15%) and flanker (5.04%) tasks. Across all tasks, 97.9% of rejected trials were flagged by channels from both amplifiers. Only the flanker task showed amplifier differences: 96% dual-flagged, 2.67% Cyton-only, 1.33% BrainAmp-only. Linear trends removed from BrainAmp and Cyton channels showed no systematic differences and were linearly correlated (see Supplementary Materials). Trial-level cross-correlation showed 98.05% perfect alignment (lag = 0), 1.8% showed a lag magnitude of 1, and no lags exceeding a magnitude of 1.

## 4. Discussion

Results indicate that Cyton and BrainAmp record ERPs with similar fidelity. Coefficients of determination ($r^{2}$) were high across subjects and conditions, and mean absolute errors (MAEs) averaged below 1 µV. Product specifications indicate a BrainAmp resolution of 0.1 µV/bit versus a Cyton resolution of 0.298 µV/bit. The largest MAE between amplifiers was 1.43 µV (subject “BC002”, flanker correct condition), which is roughly five-fold the Cyton’s resolution. Despite high $r^{2}$, low MAE, low MAAPE, and non-significant within-subject *t*-tests of amplifier means, standard deviation distributions differed significantly between amplifiers with medium-to-large effect sizes. This indicates that variation within ERP windows differs between recordings but does not affect mean amplitudes.

Noise histograms show that both amplifiers record data comparable to ERP CORE as neither significantly exceeds noise levels. Both amplifiers showed identical noise distributions, though small sample size and across-subject aggregation do not allow firm conclusions about amplifier-specific noise sensitivity. Synthetic data tests (water + sine wave generator; see Supplemental Materials) suggested that simultaneous recordings increase 60 Hz line noise relative to serial recordings, but other frequencies remain largely unaffected. Since our band-pass filter imposed a 50 Hz cutoff, increased 60 Hz noise poses minimal risk to data quality.

### 4.1. Limitations

The primary limitation is the small sample size (*n* = 5), rendering most group-level comparisons underpowered. Thus, we can only assess Cyton performance relative to the BrainAmp within individuals, not claim general population viability. Future studies should use larger samples and compare identical amplifiers (e.g., splitting electrodes across two Cytons or two BrainAmps) to establish hardware baseline variance. Although the BrainAmp supports >32 channels, the Cyton is limited to 8 channels (16 at reduced 125 Hz sampling [23]). This restricts the Cyton’s utility for high-dimensional source separation methods like independent component analysis (ICA; see [24]) or spatial filtering via current source density/common spatial patterns (see [25]). Faster sampling is possible via wired connections or custom protocols, though unsupported by official OpenBCI products.

Cross-correlation alignment poses an additional limitation. As a statistical procedure, it can miss ≥1-sample offsets due to high temporal correlation between neighboring EEG timepoints within channels. Future studies should consider dedicating an analog ExG channel to photosensor recording for precise digitization synchronization. Even with faithful photosensor recording, the OpenBCI GUI generates separate LSL streams for EEG and auxiliary data, risking misalignment in the absence of proper stream calibration.

This study examined time-domain metrics of large, well-studied ERP components. Smaller components may show larger relative amplitude differences between amplifiers. The small sample precluded latency estimates (typically computed across all subject trials [26]), though high $r^{2}$, low MAE, and low MAAPE suggest comparable latency precision. Future research should systematically compare low- and high-cost amplifiers on frequency-band and latency-specific differences.

### 4.2. Notable Issues with the OpenBCI Cyton

The OpenBCI GUI, which is the primary software for most Cyton users, is less reliable than commercial EEG software and lacks features available in other Cyton-compatible open-source alternatives. For example, it incorrectly digitizes and streams auxiliary analog data (e.g., photosensors) through LSL, which is a known issue despite reported fixes, as our experiment confirmed. Fortunately, simultaneous BrainAmp recording at higher sampling rates enabled downsampling and cross-correlation alignment. However, this approach requires simultaneous recording; synchronizing Cyton data with separately recorded electrodes from other amplifiers remains problematic—a serious current software limitation. BrainFlow offers a solution but is less user-friendly and less promoted than the GUI. OpenBCI markets the Cyton as a development board, stating that “…[it] is not considered by OpenBCI, Inc. to be a finished end-product fit for general consumer use” [23]. Yet researchers have successfully used it unmodified for over a decade.

Cyton connectivity problems can arise when other wireless devices are nearby, causing dropped packets or cross-board interference. Multi-Cyton recordings consistently fail: data collection stops (best case) or users unknowingly record from the wrong boards (worst case). A simple Faraday cage (fine metal mesh around Cyton/dongle) with a USB extension cable minimizes interference effectively.

Cyton prices have risen progressively: USD 500 → USD 1000 → USD 1250. OpenBCI’s Galea now retails at USD 36,000, far exceeding consumer range. Despite price increases, hardware quality has not improved, and the OpenBCI GUI (last updated v6.0.1 beta, September 2023) seems abandoned. This unfortunate trend undermines the Cyton’s potential for scalable neuroscience research.

### 4.3. Conclusions

Although differences exist between BrainAmp and Cyton recordings, the Cyton shows promise as a research tool, requiring further validation. It suits pilot studies, but users should compare Cyton recordings directly to “gold-standard” amplifiers under their specific recording conditions. Before deploying in naturalistic settings (schools, hospitals, homes), researchers should verify Cyton performance via simultaneous recordings with trusted amplifiers.

## Figures and Tables

**Table 1:** Trials dropped per subject due to simple voltage thresholding. Each number represents the number of trials dropped due to simple voltage thresholding. The number in parentheses represents this number as a percentage of trials dropped within the specific condition (column). Note that for the flankers task, the trials dropped represent trials removed solely from simple voltage thresholding, and not from double trial removal (see Section 2).

Subject	Frequent	Rare	Related	Unrelated	Correct	Incorrect
BC000	0 (0%)	0 (0%)	0 (0%)	0 (0%)	1 (<1%)	0 (0%)
BC001	43 (29%)	11 (18%)	0 (0%)	0 (0%)	1 (<1%)	0 (0%)
BC002	83 (56%)	20 (32%)	8 (12%)	9 (14%)	63 (17%)	3 (7%)
BC003	10 (7%)	1 (2%)	2 (3%)	3 (5%)	0 (0%)	0 (0%)
BC004	20 (14%)	2 (3%)	2 (3%)	2 (3%)	10 (3%)	0 (0%)

**Table 2:** Measures of similarity between activity recorded with the BrainAmp and Cyton. All scores were computed using the entire epoch. Arrows beside each metric represent if higher (up arrow) or lower (down arrow) values are desired. Values were computed at channels of interest for each specific event-related potential (ERP) component.

Subject	MAE (µV) ↓	MAAPE ↓	$r^{2}$↑	MAE (µV) ↓	MAAPE ↓	$r^{2}$↑
	Visual Oddball—Rare (Pz)			Visual Oddball—Frequent (Pz)		
BC000	0.76	0.2	0.99	0.75	0.2	0.99
BC001	0.68	0.19	0.99	0.69	0.2	0.99
BC002	—	—	—	—	—	—
BC003	0.54	0.17	0.99	0.54	0.17	0.99
BC004	0.84	0.21	0.98	0.8	0.22	0.98
	Word Association—Unrelated (CPz)			Word Association—Related (CPz)		
BC000	0.81	0.21	0.99	0.84	0.22	0.99
BC001	0.66	0.19	0.99	0.76	0.21	0.99
BC002	0.71	0.2	0.99	0.77	0.21	0.99
BC003	0.66	0.18	0.99	0.67	0.17	0.99
BC004	0.57	0.16	0.99	0.62	0.17	0.99
	Flankers—Incorrect (FCz)			Flankers—Correct (FCz)		
BC000	0.96	0.18	0.99	1.01	0.2	0.99
BC001	0.78	0.19	0.99	0.81	0.2	0.99
BC002	1.39	0.27	0.97	1.43	0.27	0.98
BC003	0.6	0.15	1.0	0.64	0.19	0.99
BC004	0.89	0.19	0.99	0.85	0.19	0.99

**Table 3:** Effect sizes (Cohen’s d) of standard deviation measurements within ERP windows of interest. Each value was computed at the channel of interest, indicated in parentheses in the column name. Each asterisk denotes an order of magnitude increase in significance, with a baseline, Bonferroni-corrected *p* value of 0.0025 being one asterisk. ns = non-significant. Subject “BC002” was excluded from P300 analyses due to having too many artifactual trials. These data are derived from data also shown in the lower portion of Figure 3.

	Visual Oddball (Pz)		Word Association (CPz)		Flankers (FCz)	
	Rare	Frequent	Unrelated	Related	Incorrect	Correct
Subject	d	d	d	d	d	d
BC000	−1.38 ***	−1.64 ***	−1.11 ***	−1.04 ***	−0.69 ***	−1.06 ***
BC001	−1.59 ***	−1.58 ***	−1.27 ***	−1.21 ***	−0.69 ***	−1.16 ***
BC002	—	—	−1.83 ***	−1.96 ***	−1.04 ***	−1.57 ***
BC003	−1.44 ***	−1.29 ***	−0.66 ***	−0.95 ***	ns	−0.88 ***
BC004	−1.16 ***	−1.00 ***	−1.11 ***	−1.33 ***	−0.50 *	−0.73 ***

## Data Availability

All data, experiment files, and analysis scripts will be made available upon publication.

## Supplementary Materials

Additional methods are provided in the Supplementary Materials that can be downloaded at: https://www.mdpi.com/article/10.3390/s26041153/s1. Reference [27] is cited in the supplementary materials.
