# Supplementary material for: Comparing Simultaneous Scalp EEG Recordings from the OpenBCI Cyton and Brain Products BrainAmp
Source: Sensors (Basel). 2026 Feb 11;26(4):1153. doi: 10.3390/s26041153 (PMC12944456; doi:10.3390/s26041153)
Supplement: Supplementary file 1 [file sensors-26-01153-s001.zip › sensors-4087958-supplementary.pdf]

Comparing Simultaneous Scalp EEG Recordings from the OpenBCI Cyton and Brain Products

Alessandro D'Amico and Virginia R. de Sa

February 10, 2026

1 Methods

1.0.1 Flankers Task

In the flankers task, there was an inter-trial interval between 1,200 and 1,400 milliseconds, and each arrow was approximately $1 \times 1^\circ$ in size. Stimuli were presented for approximately 200 milliseconds. Participants completed 10 blocks of 40 trials. Participants were instructed to respond with the arrow key corresponding to the pointing direction of the central arrow; either left or right. Analyses were conducted on an epoch spanning [-600, 400] ms locked to response onsets. The window containing the components of interest spanned [0, 100] ms, and the epoch was baseline corrected using the average of activity in the window spanning [-400, -200] ms.

1.0.2 Word Association Task

For the word association task, prime words were presented in red (RGB 255, 0, 0), while target words were presented in green (RGB 0, 255, 0). The inter-stimulus interval between prime and target words was between 900 and 1,100 milliseconds, while the inter-trial interval was between 1,400 and 1,600 milliseconds. Words were presented for approximately 200 milliseconds. Each letter was approximately $1 \times 1^\circ$ in size. Participants completed 6 blocks of 20 trials each. Each sentence had a congruent and incongruent ending. Participants saw only one of these endings within the first three blocks, and would see the other ending in the last three blocks. Participants indicated whether a pairing was congruent or incongruent using the up or down arrow keys. Analyses were conducted on an epoch spanning [-200, 800] ms locked to target

stimulus onsets. The window containing the components of interest spanned [300, 500] ms, and the epoch was baseline corrected using the average of activity in the window spanning [-200, 0] ms.

1.0.3 Oddball Task

The oddball task deviated slightly from ERP CORE. Rather than having 200 trials spread over 5 blocks, participants completed 10 blocks of 20 trials each. Each block either had a target rate of 20%, or 40%, which were balanced and randomized within subjects. The reason for this modification was to replicate the well-observed inverse relationship between the probability of the target stimulus and the amplitude of P3b (Duncan-Johnson & Donchin, 1977). However, this change in P3b amplitude as a result of target probability is not examined in this work, and all target trials were combined for analysis. Otherwise, parameters were consistent with ERP CORE; stimuli were presented for approximately 200 milliseconds, with an inter-trial interval between 1,200 and 1,400 milliseconds. Each stimulus was a single letter (A, B, C, D, or E), with one letter being selected as the target, and the other nontargets. Letters were approximately $2.5\times2.5^\circ$ in size. Participants indicated whether a letter was a target or nontarget using the up or down arrow keys. Analyses were conducted on an epoch spanning [-200, 800] ms locked to letter stimulus onsets. The window containing the components of interest spanned [300, 600] ms, and the epoch was baseline corrected using the average activity in the window spanning [-200, 0] ms.

1.1 Linear Trend Analysis

During qualitative analysis of pilot data, a linear trend was observed in OpenBCI Cyton recordings. Grand average ERPs looked to have a slightly different linear trend between the BrainAmp and Cyton electrodes. This linear trend was efficiently removed with a high-pass filter described in the main text. In order to examine if there were systematic differences between the slopes of the linear trends between the amplifiers, we plotted the slope of each epoch from the BrainAmp and Cyton against each other pooled across all subjects, paradigms, conditions, and channels after filtering. Overall, there does not appear to be any systematic linear trend differences between the amplifiers after filtering (see Figure S1).

1.2 Synthetic Signal Comparisons

As a secondary measurement of comparison and one that is independent of human subjects, tests were performed on data collected in saline solution by a sine wave generator. In these tests, the same electrodes

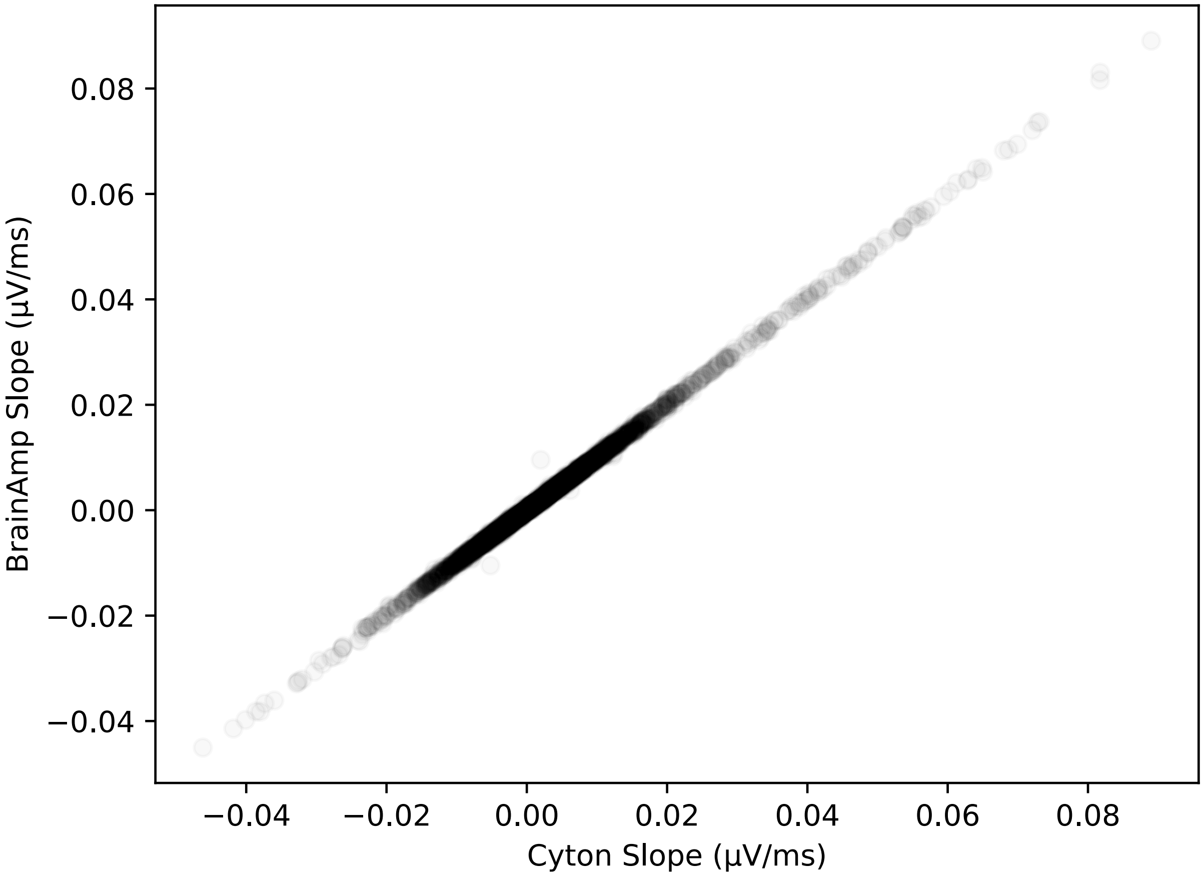

Figure S1: Linear plot comparing Cyton and BrainAmp slopes after filtering. Slopes were fitted independently for each channel within each epoch. Each point has an opacity of 3% in order to highlight the cluster between -0.02 and 0.02 $\frac{\mu V}{ms}$ between both amplifiers.

that were used in the human subject recordings were placed in a solution of table salt (NaCl, including iodine) and tap water. Three electrodes were used: ground, reference, and a recording electrode. A signal generator (15 MHz DDS Arbitrary Waveform Function Generator made by Koolertron) had leads placed in the same basin such that the positive and negative terminals were on opposite sides of the circle's diameter. The reference electrode was placed beside the negative output of the function generator, and the recording electrode was placed beside the positive output of the function generator. The ground electrode was placed in the center of the circular basin. In order to minimize the influence of 60 Hz line noise, the signal generator was powered by a 3.7V 20,000 mAh battery.

We recorded sine waves from the function generator using a simple grid search with three different voltages and frequencies, resulting in nine trials per recording. This was repeated with recordings of only the Cyton, only the BrainAmp, and with the electrodes Y-split into both amplifiers, mirroring the human subject recording. Additionally, these tests were performed with active electrodes designed for the Brain Vision amplifiers.

The signals recorded from saline are generally quite different from those recorded from human skin. Electrodes placed on human skin have nonlinear capacitive effects, so saline tests don't capture the true characteristic of these purpose-made bioamplifiers. They do provide an interesting opportunity to qualitatively compare the signals recorded by the amplifiers simultaneously and independently in a repeatable manner, however, which cannot be done with the human subjects. Power spectral densities were computed for each trial and served as the basis for qualitative analyses.

Qualitative analyses suggest that simultaneous recordings do not significantly alter the recorded signals (see Figures S4 and S5), and that activity in stimulated frequencies is fairly similar between the BrainAmp and the Cyton (see Figure S3). However, we do observe an increase in 60 Hz line noise in simultaneous recordings. This is likely caused by the fact that additional unshielded cables (the Y-splitter) are used in order to send the signals into both amplifiers. We also note that the Cyton generally has lower power around 60 Hz compared to the BrainAmp. Active electrodes don't appear to have any effect in these recordings (see Figure S2), although as noted earlier, these saline tests are not accurate indicators of the full system's performance.

References

Duncan-Johnson, C. C., & Donchin, E. (1977). On quantifying surprise: The variation of event-related potentials with subjective probability. *Psychophysiology*, *14*(5), 456–467.

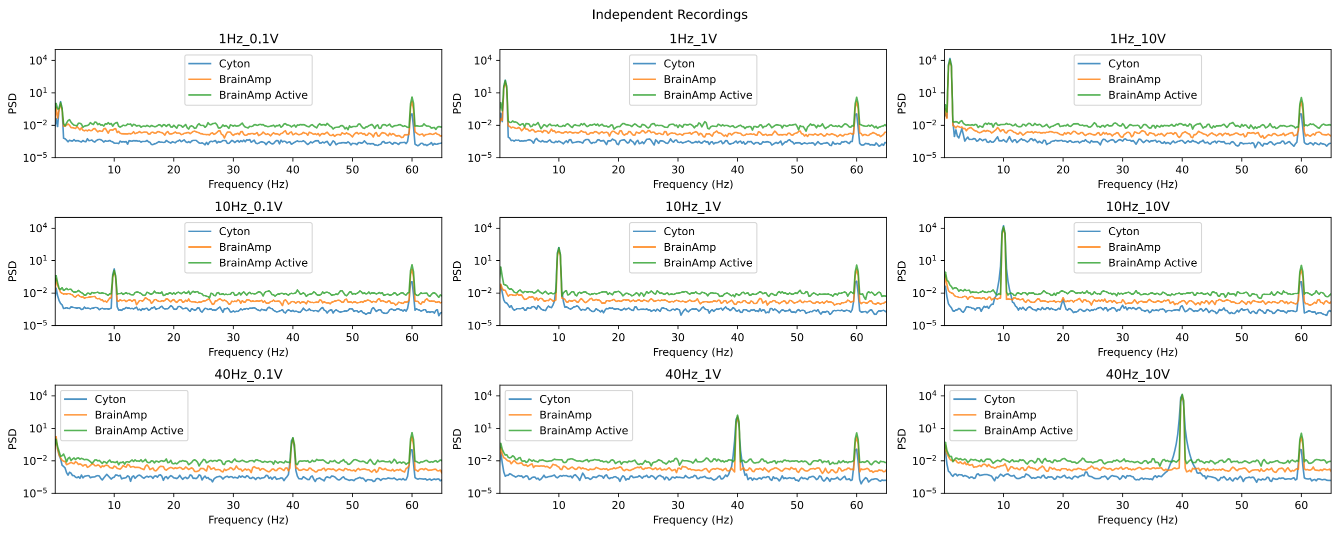

Figure S2: Traces from independent recordings. Each of the traces within the subplots was recorded independently of all other traces. The frequencies listed in each subplot's title are accurate, but the voltages are not. The voltage listed was the voltage supplied by the function generator, but this was stepped down using variable resistance. PSDs are in units $\frac{\mu V^2}{Hz}$. Note that the same electrodes used on the human EEG data were used for the Cyton and BrainAmp recordings, but the BrainAmp Active recordings utilize a different set of electrodes. The active electrodes are those most commonly used with the BrainAmp, thus its inclusion in this comparison. A notable conclusion is that the stimulated frequencies exhibit the same power across all recording modalities. 60 Hz line noise is very similar in both passive and active BrainAmp recordings. 60 Hz line nose appears lower in the Cyton than in the BrainAmp.

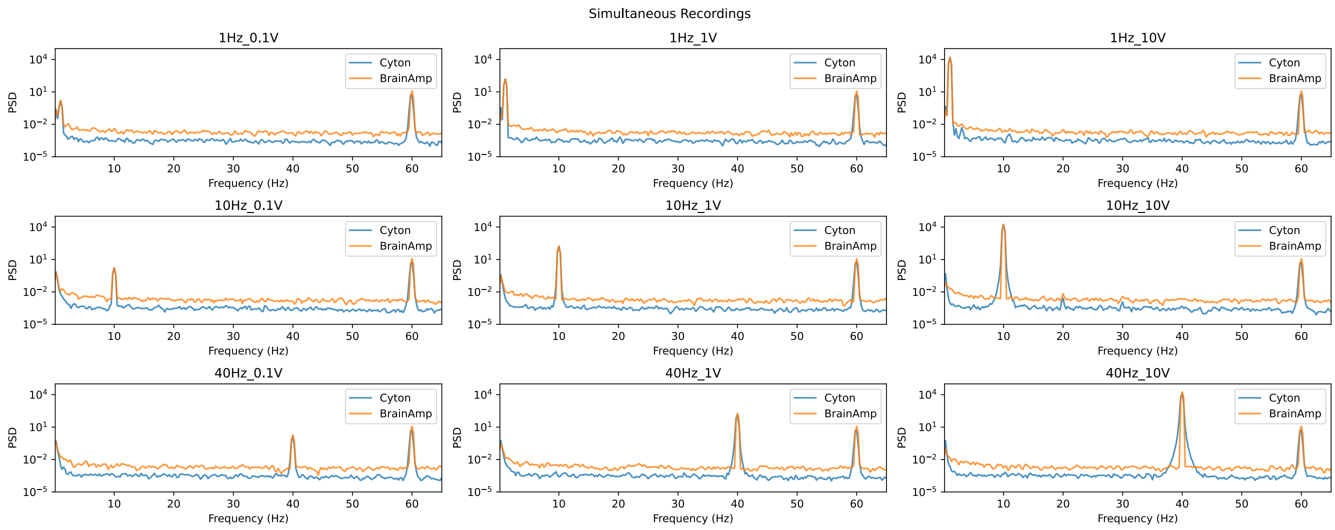

Figure S3: Traces from simultaneous recordings. One set of electrodes was Y-split into both amplifiers in a way identical to the human EEG recordings. The frequencies listed in each subplot's title are accurate, but the voltages are not. The voltage listed was the voltage supplied by the function generator, but this was stepped down using variable resistance. PSDs are in units $\frac{\mu V^2}{Hz}$. A notable conclusion is that stimulated frequencies and 60 Hz bands are similar between both amplifiers.

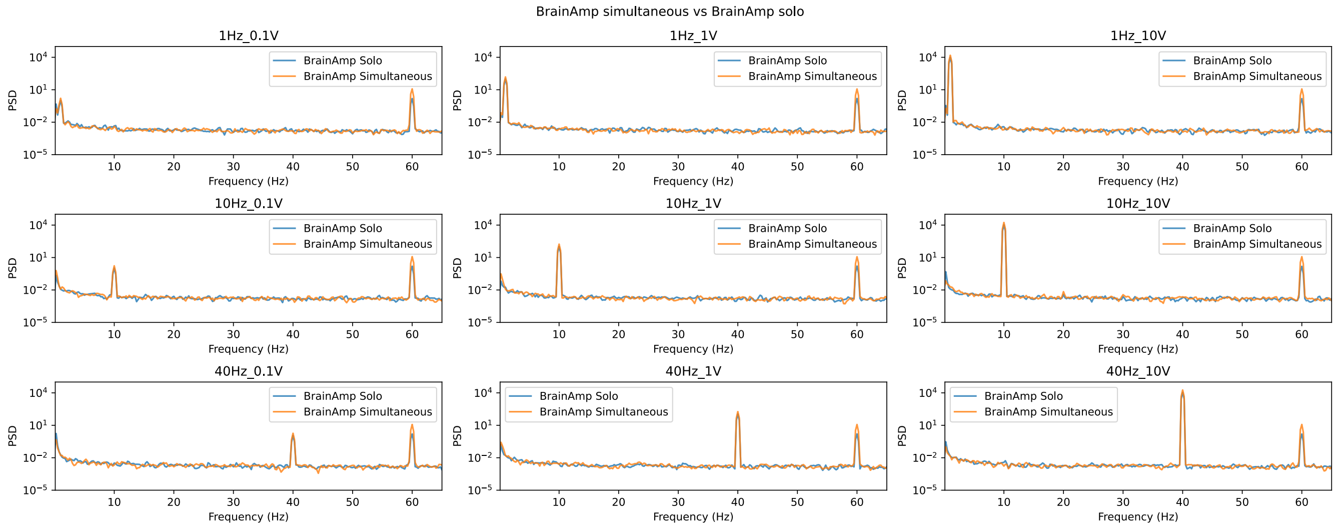

Figure S4: Traces from independent (solo) and simultaneous recordings from the BrainAmp. These traces are seen in earlier figures S3 and S2, but only the BrainAmp traces are shown together. The frequencies listed in each subplot's title are accurate, but the voltages are not. The voltage listed was the voltage supplied by the function generator, but this was stepped down using variable resistance. PSDs are in units $\frac{\mu V^2}{Hz}$. A notable conclusion is that stimulated frequencies and the background bands are similar, yet there is a notable increase in 60 Hz line noise resulting from the simultaneous recording. This is likely due to an increase in unshielded passive cabling length required to facilitate the simultaneous recordings.

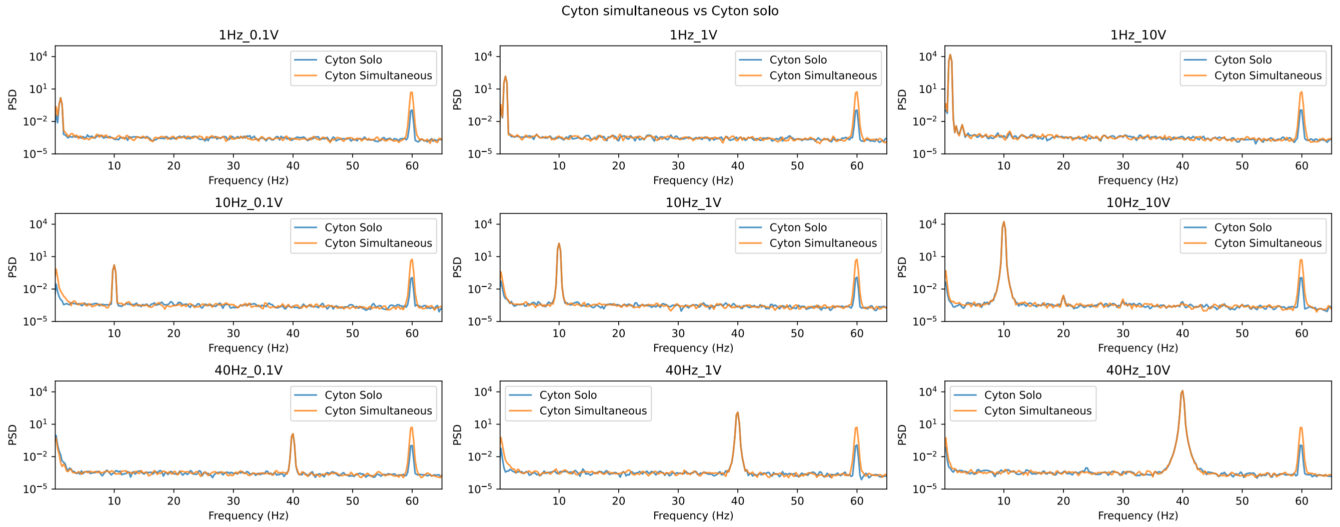

Figure S5: Traces from independent (solo) and simultaneous recordings from the Cyton. These traces are seen in earlier Figures S3 and S2, but only the Cyton traces are shown together. The frequencies listed in each subplot's title are accurate, but the voltages are not. The voltage listed was the voltage supplied by the function generator, but this was stepped down using variable resistance. PSDs are in units $\frac{\mu V^2}{Hz}$. A notable conclusion is that stimulated frequencies and the background bands are similar, yet there is a notable increase in 60 Hz line noise resulting from the simultaneous recording. This is likely due to an increase in unshielded passive cabling length required to facilitate the simultaneous recordings.
